# An opportunity to emphasize the relevance of laboratory medicine

**DOI:** 10.1515/almed-2021-0029

**Published:** 2021-07-09

**Authors:** María Santamaría González, María Ángels Ruiz Mínguez, María Monsalud Arrebola Ramírez, Xavier Filella Pla, María José Torrejón Martínez, Daniel Morell García, Miguel Ángel Castaño López, Juan Antonio Allué Palacín, María Dolores Albaladejo Otón, Nuria Giménez Gómez

**Affiliations:** Service of Clinical Biochemistry, Miguel Servet University Hospital, Zaragoza, Spain; Commission of Evidence-Based Laboratory Medicine, Spanish Society of Laboratory Medicine (SEQC^ML^), Barcelona, Spain; Service of Laboratory Medicine, Fundació Hospital de l’Esperit Sant, Santa Coloma de Gramenet, Barcelona, Spain; Laboratory Clinical Management Unit, Hospital de la Axarquía (AGSEMA), Málaga, Spain; Service of Biochemistry and Molecular Genetics (CDB), Hospital Clinic, IDIBAPS, Barcelona, Spain; Clinical Biochemistry Management Unit (UGC), Hospital Clínico San Carlos, Madrid, Spain; Service of Laboratory Medicine, Hospital Universitari Son Espases, Palma de Mallorca, Spain; Service of Clinical Biochemistry, Hospital Clínico Universitario Juan Ramón Jiménez, Huelva, Spain; Synlab Diagnosticos Globales, Sevilla, Spain; Service of Laboratory Testing and Clinical Biochemistry, Santa Lucía University Hospital, Cartagena, Spain; Research Unit, Research Foundation, Mutua de Terrassa, University of Barcelona, Barcelona, Spain; Laboratory of Toxicology, Universitat Autònoma de Barcelona, Barcelona, Spain; Commission of Evidence-Based Laboratory Medicine, International Federation of Clinical Chemistry (IFCC), Milan, Italy

**Keywords:** biomarkers, clinical laboratory, clinical practice guidelines, evidence-based medicine, laboratory medicine

## Abstract

**Objectives:**

Clinical practice guidelines (CPGs) are recommendations based on a systematic review of scientific evidence that are intended to help healthcare professionals and patients make the best clinical decisions. CPGs must be evidence-based and are designed by multidisciplinary teams. The purpose of this study is to assess the topics related to the clinical laboratory addressed in CPGs and evaluate the involvement of laboratory professionals in the CPG development process.

**Methods:**

A total of 16 CPGs recommended by the Spanish Society of Laboratory Medicine and/or retrieved from PubMed-Medline were included. A review of the information provided in CPGs about 80 topics related to the clinical laboratory was performed. The authorship of laboratory professionals was assessed.

**Results:**

On average, the 16 CPGs addressed 49% (standard deviation [SD]: 11%) of the topics evaluated in relation to the clinical laboratory. By order of frequency, CPGs contained information about 69% of postanalytical variables (SD: 20%); 52% of preanalytical variables (SD: 11%); and 43% of the analytical variables studied (SD: 18%). Finally, half the CPGs included a laboratory professional among its authors.

**Conclusions:**

CPGs frequently failed to provide relevant laboratory-related information. Laboratory professionals were co-authors in only half the CPGs. There is scope for improvement, and laboratory professionals should be included in multidisciplinary teams involved in the development of CPGs.

## Introduction

Clinical practice guidelines (CPGs) are defined as “*recommendations informed by a systematic review of evidence and an assessment of the benefits and costs of alternative care options intended to optimize patient care*” [1]. They are one of the most important tools for the optimization of clinical decisions [2].

CPGs for a specific condition must be developed by multidisciplinary teams composed of clinicians involved in the management of the disease. CPGs should be based on a scientific, rigorous, and transparent methodology and establish recommendations informed by the best evidence available [3]. In clinical practice, there is some confusion about the difference between CPGs and clinical protocols. Protocols are documents detailing the sequence of processes to be applied for the management of a specific health problem. They have a regulatory nature, are agreed and consider the resources available in the center where they are intended to be applied [4], whereas CPGs are at the top of the hierarchical pyramid of sources of evidence [5].

Since 2004, the Commission of Evidence-Based Laboratory Medicine of the Spanish Society of Laboratory Medicine (SEQC^ML^-) has coordinated a workgroup that disseminates the CPGs of the highest quality and/or its impact on clinical practice based on the opinions of the different SEQC^ML^- Commissions. This way, this commission contributes to the dissemination of quality scientific information.

It is worth mentioning that CPGs frequently fail to provide quality information about laboratory testing. A recent study assessing CPGs about prostate cancer screening with the prostate-specific antigen revealed that a laboratory professional had been involved in the development of only 9% of CPGs, and relevant information about laboratory tests was missing [6]. Surprisingly, a laboratory professional was not included in most multidisciplinary teams developing CPGs, even in those where laboratory testing plays an essential role in the management of a specific health problem.

The purpose of this study is to review the information related to the clinical laboratory contained in CPGs when recommendations for use of diagnostic and follow-up laboratory tests are included and evaluate the level of involvement of laboratory professionals in the CPGs’ development process.

## Materials and methods

### Source of information and search strategies

A retrospective, descriptive, observational study was carried out to review CPGs published in the last five years (2015–2019). CPGs were retrieved from two sources of information: the website of the SEQC^ML^, visited in December 2019, and the repository of health and medical literature PubMed.

The CPGs recommended by SEQC^ML^ scientific commissions are available at http://www.seqc.es/es/gpc/.

The literature search was limited to papers related to laboratory medicine published in the last five years: ((“Guideline” [PublicationType] OR “Guidelines as Topic”[Mesh] OR guideline*) AND (“Clinical Laboratory Services”[Mesh] OR “Laboratories”[Mesh] OR laboratories)) AND (“Biomarkers”[Mesh] OR biomarkers) (search performed on November 29, 2019).

### Inclusion criteria

CPGs were candidates for inclusion if they were guidelines, had been published in the previous five years, were written in English, and included recommendations for use of laboratory tests for diagnostic or therapeutic purposes. Redundant CPGs were only included once.

### Data collection

CPGs were selected by two independent reviewers who applied the inclusion criteria described above. Discrepancies were solved by a third reviewer.

The CPGs selected were evaluated by peer review. The reviewers searched for relevant clinical laboratory data related to preanalytical, analytical, and postanalytical processes that may influence the clinical interpretation of test results. To such a purpose, we applied the checklist including 80 topics that should be considered for all laboratory tests included in a clinical practice guideline proposed by Aakre et al. [7] and adapted to the CPGs reviewed. Each study variable was converted into a dichotomous YES (1) or NO (0) variable, with being 0 the lowest score and 80 the highest.

The involvement of a laboratory medicine specialist in the guideline development process was assessed based on author affiliations. ‘*Laboratory medicine specialist*’ was defined as any professional with a specific university degree, with authority to exercise a specific function, and final personal accountability, who develops their professional practice in a laboratory, whether it was clinical or research practice.

### Statistical analysis

Categorical variables are expressed as percentages. Normality of distribution was assessed by the Kolmogorov-Smirnov and Shapiro-Wilk tests. Continuous variables are expressed as a central tendency (mean or median) and dispersion standard deviation (SD) or interquartile range (IQR), based on their distribution. Qualitative variables were compared using χ^2^. Comparison of quantitative variables was performed by Student’s t-test or the nonparametric Mann-Whitney U-test. All differences with a p-value of 0.05 were considered statistically significant. Statistical analyses were performed using IBM SPSS version 25 (Armonk, NY, USA).

## Results

**Figure 1:**
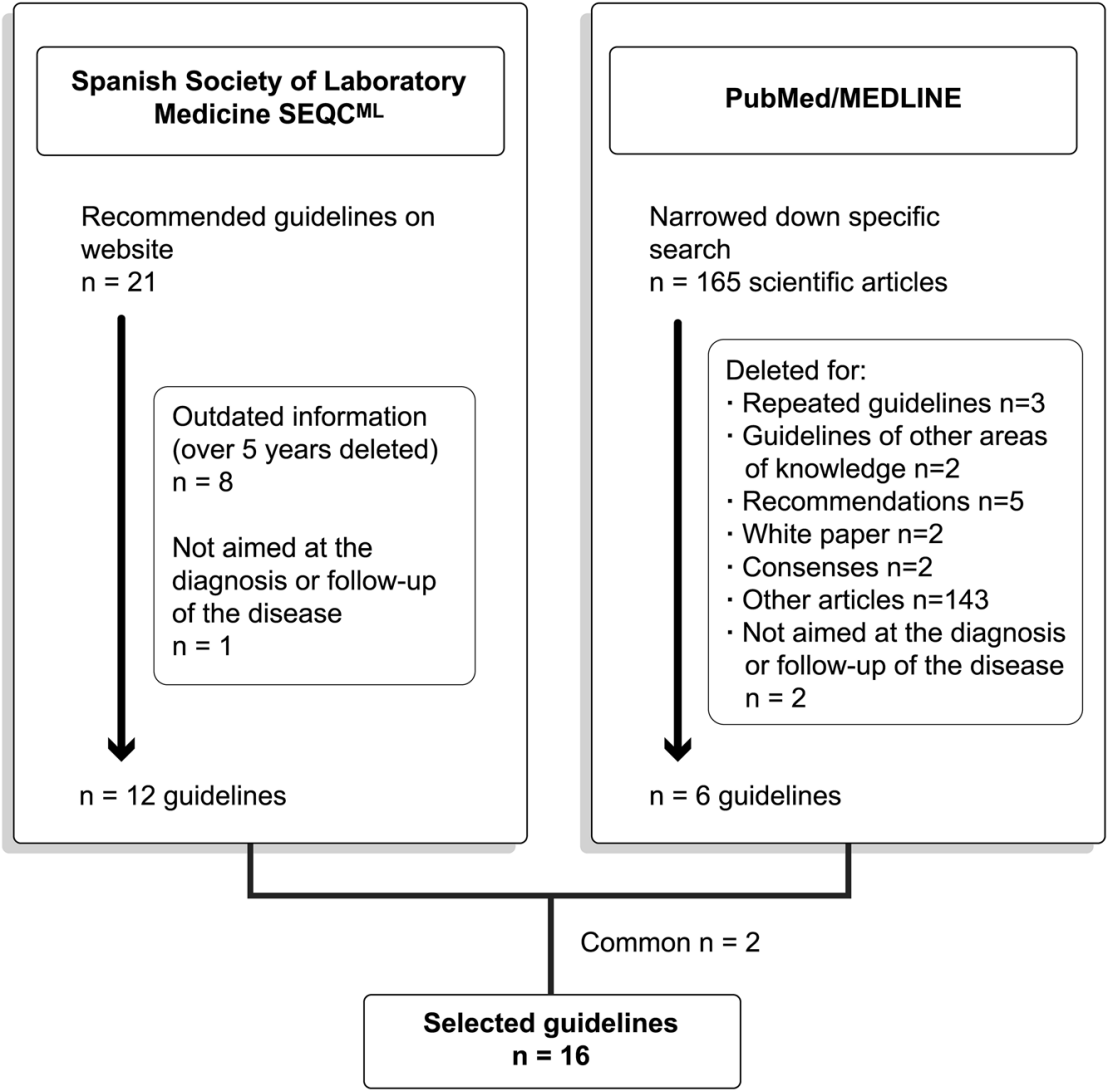
Clinical practice guideline selection process.

The CPGs selection process is displayed in Figure 1. A total of 165 CPGs were retrieved from PubMed, of which six met the inclusion criteria. The 143 papers that were not clinical practice guidelines but made reference to CPGs were excluded. A revision was performed of the 12 CPGs recommended by the SEQC^ML^ that met the inclusion criteria. Finally, 16 CPGs were selected for revision and evaluation (Table 1).

**Table 1:** Clinical practice guidelines (CPGs) reviewed and percentage of CPGs that provide relevant data about the preanalytical, analytical and postanalytical phase.

CPG reviewed (n=16) (Maximum score possible)	Phases of the analytical process^a^				Laboratory professional n=16
	Preanalytical (33 points)	Analytical (38 points)	Postanalytical (9 points)	Total (80 points)	
Clinical use of cancer biomarkers in epithelial ovarian cancer: updated guidelines from the European Group on Tumor Markers [8].	50% (16/32)	13% (5/38)	78% (7/9)	35% (28/79)	Yes
Clinical use of biomarkers in breast cancer: updated guidelines from the European Group on Tumor Markers [9].	46% (12/26)	44% (11/25)	50% (3/6)	46% (26/57)	Yes
Revised American Thyroid Association Guidelines for the management of medullary thyroid carcinoma [10].	61% (17/28)	68% (15/22)	78% (7/9)	66% (39/59)	Yes
Congenital adrenal hyperplasia due to steroid 21-hydroxylase deficiency: An Endocrine Society Guideline [11].	66% (21/32)	68% (15/22)	67% (6/9)	67% (42/63)	No
Clinical practice guidelines for the care of girls and women with turner syndrome [12].	42% (11/26)	73% (8/11)	60% (3/5)	52% (22/42)	Yes
European Guidelines on cardiovascular disease prevention in clinical practice [13].	48% (12/25)	52% (15/29)	56% (5/9)	51% (32/63)	No
Guideline on the management of blood cholesterol [14].	48% (16/33)	42% (16/38)	89% (8/9)	50% (40/80)	Yes
Guidelines for the management dyslipidaemias [15].	58% (19/33)	45% (17/38)	89% (8/9)	55% (44/80)	Yes
Guidelines for the diagnosis and treatment of acute and chronic heart failure [16].	27% (9/33)	18% (7/38)	56% (5/9)	26% (21/80)	No
Guidelines for the management of acute coronary syndromes in patients presenting without persistent ST-segment elevations [17].	76% (22/29)	33% (11/33)	89% (8/9)	58% (41/71)	No
Chronic kidney disease in adults [18].	42% (14/33)	39% (15/38)	89% (8/9)	46% (37/80)	No
Anemia management in chronic kidney disease [19].	69% (20/29)	42% (13/31)	89% (8/9)	59% (41/69)	No
Updated molecular testing guideline for the selection of lung cancer patients for treatment with targeted tyrosine kinase inhibitors [20].	55% (18/33)	13% (5/38)	44% (4/9)	34% (27/80)	Yes
Biomarkers in nonsmall cell lung cancers: Indian consensus guidelines for molecular testing [21].	48% (14/29)	53% (17/32)	89% (8/9)	56% (39/70)	Yes
ESMO consensus guidelines for the management of patients with metastatic colorectal cancer [22].	45% (10/22)	37% (10/27)	22% (2/9)	38% (22/58)	No
Human epidermal growth factor receptor 2 testing in breast cancer [23].	48% (11/23)	48% (10/21)	56% (5/9)	49% (26/53)	No
**Total**	52%	43%	69%	49%	50% (8/16)

^a^Results are expressed as % (absolute number of addressed topics related to the laboratory/absolute number of relevant laboratory topics).

All phases of the analytical process were adequately represented in the CPGs reviewed which, on average, addressed 49% (SD: 11%) of the clinical laboratory topics evaluated in the study. By order of frequency, 69% (SD: 20%) of the postanalytical variables, 52% (SD: 11%) of preanalytical variables, and 43% (SD: 18%) of the analytical variables studied were addressed in the CPGs.

The information provided in CPGs about each phase is detailed in Table 2 by the involvement of laboratory medicine specialists and by the source of information. A laboratory professional was involved in the development of 50% of the CPGs. An association was not observed between the laboratory-related information contained in a CPG and the involvement of a laboratory professional in the development process (p=1.000). The source of information from which the CPG was selected was not associated with the laboratory topics addressed.

**Table 2:** Topics addressed in clinical practice guidelines related to the different analytical phases by authorship and source of information.

Information provided in clinical practice guidelines (% = number of laboratory topics addressed/maximum number of topics)			
CPGs developed with the involvement of laboratory medicine specialists.			
n=16	Yes (n=8)	No (n=8)	p-Value
Preanalytical phase. Mean (SD)^a^	51(6)	53(16)	0.799
Analytical phase. Mean (SD)^a^	44 (22)	42 (15)	0.859
Postanalytical phase. Median (IQR)^b^	78 (37)	62 (33)	0.705
Total analytical process. Mean (SD)^a^	49 (11)	49 (13)	1.000
**Source of information**			
**n=14** ^**d**^	**SEQC-ML (n=10)**	**PubMed (n=4)**	**p-Value**
Preanalytical phase. Mean (SD)^a^	54 (15)	49 (4)	0.382
Analytical phase. Mean (SD)^a^	48 (17)	38 (18)	0.342
Postanalytical phase. Median (IQR)^b^	84 (30)	50 (53)	0.089
Total analytical process. Mean (SD)^a^	53 (12)	44 (10)	0.214
Authorship of laboratory medicine specialists^c^	40%	67%	0.298

SD, standard deviation; IQR, interquartile range. ^a^Student’s t-test. ^b^Nonparametric Mann-Whitney U-test. ^c^χ^2^-test. ^d^Repeated CPGs were considered only once for statistical analysis.

Table 3 summarizes the frequencies of appearance of the 80 topics investigated. In relation to the preanalytical phase, aspects related to sample requirements were most frequently missing. In the analytical phase, the least frequently addressed topic was the presence of analytical interferences.

**Table 3:** Checklist employed to review clinical practice guidelines (CPGs) (modified by Aakre et al. [7]).

Preanalytical phase		CPG inclusion
Description of the target population	Age	88%
	Sex	84%
	Disease	96%
	Specific diseases	94%
Indications for biomarker use	Monitoring	84%
	Frequency of testing	68%
	Diagnosis	83%
	Prognosis	88%
	Screening	57%
	Auto-monitoring	50%
Clinical performance	Sensitivity	65%
	Specificity	59%
	ROC curve	6%
	Added value of the biomarker	81%
	Comparison with other related biomarkers	69%
	Probability of diagnosis after the test	52%
	Positive outcome of testing	31%
	Negative outcome of testing	28%
Multiple approach with other biomarkers	Inclusion in a panel with other biomarkers	71%
	Sensitivity (panel)	39%
	Specificity (panel)	39%
	ROC curve (panel)	0%
	Added value of the panel	57%
Sample requirements	Fasting	35%
	Time from the clinical event	28%
	Patient position	5%
	Circadian rhythm	10%
	Type of sample	75%
	Sample transportation	9%
	Centrifugation	0%
	Sample pretreatment (maximum delay)	19%
	Maximum time of storage at the specified temperature	6%
	Maximum no. of freezing-thawing cycles	3%

Analytical phase		CPG inclusion
Method	Recommended method	72%
	Standardization	41%
	Traceability to the method of reference	38%
	Heterogeneity of biomarkers	50%
	Limit of detection	17%
Analytical interferences	Lipemia	6%
	Hemolysis	0%
	Bilirubin	0%
	Monoclonal paraproteins	6%
	Heterophile antibodies	16%
	Endogenous antibodies	15%
	Rheumatoid factor	6%
	Other relevant	30%
	Define actions on suspicion of analytical interference	10%
Biological interferences	Age	75%
	Sex	68%
	Acute disease	70%
	Acute phase	59%
	Food intake	42%
	Medication	52%
	Tobacco	33%
	Alcohol	30%
	Pregnancy	29%
	Postmenopause	18%
	Obesity	27%
	Physical activity	38%
	Genetic factors	60%
	Ethnicity	37%
	Geographic region	37%
	Other	47%
Qualty issues	Analytical variability	53%
	External quality assessment	50%
	Internal quality assessment	44%
	Criteria/Quality goals	25%
	Bias, imprecision, total error	22%
	Accreditation	28%
	Personnel education and training	31%
	Turnaround time	31%
**Postanalytical phase**		**CPG inclusion**
	Quantitative or qualitative results	94%
	Units	87%
	Comments about results	56%
	Reference interval	48%
	Diagnostic cut-off point	90%
	Therapeutic goal	88%
	Biological variability	41%
	Information about significant clinical changes (RCV)	37%
	Interpretation about changes based on RCV or clinical study results	50%

Contains 80 relevant topics related to the clinical laboratory distributed across the preanalytical, analytical and postanalytical phase. If a topic was not considered relevant to the evaluation of the CPGs, the nominator was adjusted for percentage calculation.

## Discussion

This study demonstrates that, despite the relevance of CPGs for the optimization of patient care, there is scope for improvement in the clinical laboratory. Thus, laboratory medicine specialists should be invited to join multidisciplinary guideline development teams. In addition, more detailed information should be provided in CPGs about specific aspects of diagnostic laboratory tests, which are essential in clinical decision-making [24–26].

The CPGs including recommendations for use of laboratory tests for diagnostic or follow-up purposes only provided half the relevant information related to laboratory testing. Moreover, specific aspects such as preanalytical sample requirements or analytical interferences were hardly mentioned. On the other hand, all analytical phases i.e. preanalytical, analytical, and postanalytical, were considered. It is important to be aware of the limitations and potential errors that may occur across the different laboratory testing processes, as they may have a negative impact on the patient [27]. Laboratory testing is an essential part of clinical decision-making, and most CPGs include recommendations for use of laboratory tests. However, information about some relevant aspects of laboratory processes is missing [6, 7] despite being necessary for an appropriate interpretation of results [27]. The quality of CPGs could be improved by addressing relevant aspects of laboratory processes [6, 7].

A laboratory professional had been involved in the development of half the CPGs studied, which is a low rate, considering that we selected CPGs that recommended specific laboratory tests for diagnosis and follow-up of disease. Surprisingly, the rate of participation of laboratory professionals in the CPG development process was even lower in the few studies where this topic was addressed [6, 7]. All the professionals involved in the management of a specific disease should take part in the development of CPGs for that disease, which would guarantee multidisciplinarity. It is likely that relevant aspects about laboratory testing are not appropriately addressed when a laboratory professional hasn’t taken part in the recommendations for use of a laboratory test in a guideline [28]. Additionally, the quality of CPGs would improve if evidence about the clinical relevance of laboratory tests was provided, which would also contribute to optimize the use of the clinical laboratory and the usefulness of new biomarkers [24]. The complexity of laboratory testing demands a good understanding of CPGs and the knowledge of the limitations of the analytical methods by laboratory staff, who also help interpreting and using laboratory data [28]. The activity of laboratory professionals is interconnected with all medical disciplines and provides crucial support in healthcare [26]. Therefore, it is essential to raise awareness about the relevance of clinical advice and the involvement of laboratory professionals in patient care. Moreover, laboratory medicine specialists should more frequently join the multidisciplinary teams that develop, review, disseminate, and use CPGs.

Paradoxically, unlike previous studies, in our study, the involvement of laboratory medicine specialists in the guideline development process did not lead to the increased attention given to laboratory-related topics [7, 28]. Thus, Akree et al. documented that more information is provided about the type of sample, shipping, and other analytical aspects in the CPGs developed by a multidisciplinary team, including a laboratory medicine specialist [7]. The British Thoracic Society guidelines for the investigation of unilateral pleural effusion in adults also recommend a resource to laboratory medicine specialists for improving the use and interpretation of laboratory tests by clinicians [28]. No statistically significant differences were observed in laboratory-related information quality and the involvement of a laboratory medicine specialist in the CPG development process. This may be due to our selection method. The sample was composed of high-quality CPGs recommended by the SEQC^ML^ Commission for their relevance and usefulness for the clinical laboratory or retrieved from a literature search on PubMed limited to clinical laboratory topics. We postulate that although the authorship of laboratory professionals is not recognized in a CPG, they may have been involved in the development process as external collaborators, advisers, expert consultants, and/or reviewers, as many aspects related to laboratory testing are addressed in these CPGs.

Laboratory medicine professionals are specialists in the biochemical and biological interactions that characterize human diseases. Their professional experience with laboratory testing and its clinical relevance position empowered them as reliable consultants with an in-depth knowledge of preanalytical requirements and of the clinical relevance and interpretation of test results. Hence, laboratory professionals provide supplementary information to the one recorded in medical reports and guarantee quality patient care [29]. Additionally, they contribute to reduce potential diagnostic errors and improve the quality of healthcare and health services [30]. Laboratory medicine can be occasionally perceived as a neglected discipline, which is clearly seen in the high proportion of papers showing a lack of understanding about the relevance of laboratory testing [25]. Laboratory medicine specialists should increase their visibility and get more actively involved in clinical teams. This would raise awareness about the relevance of their role and the added value they can bring as consultants in patient-centered care.

SEQC^ML^, as a scientific society, prioritizes access to high-quality information and recommends CPGs grouped by fields of medicine. These CPGs are regularly updated to incorporate recent evidence and are intended to meet the needs of laboratory medicine specialists. Health professionals find it difficult to cope with the flood of biomedical information published every month since searching for high-quality publications is time-consuming [31]. The availability of a website providing access to relevant CPGs facilitates the selection of quality publications by laboratory professionals [32]. Thus, scientific societies meet one of their main goals: managing and promoting the generation, uptake, dissemination, and handling of knowledge [33]. The publication of CPGs recommended by the SEQC^ML^ on its website is complemented by other multiple initiatives launched in collaboration with other scientific societies and raises the visibility of the role of laboratory medicine specialists.

Thus, with the publication of quality CPGs on its website, the SEQC^ML^ facilitates the translation of CPGs recommendations into clinical practice, including laboratory medicine practice [34], hence giving easy, rapid access to CPGs previously reviewed and organized by topics. The need to promote the implementation of CPGs by laboratory medicine specialists is recognized by the scientific community, and the benefits of their involvement in CPG development and implementation are widely known [35]. However, laboratory medicine specialists have difficulties to access and join multidisciplinary CPG development teams.

The reduced sample of CPGs reviewed may be a limitation of this study. We retrieved all CPGs recommended by the SEQC^ML^ and a literature search of CPGs addressing topics related to the clinical laboratory was performed. Therefore, despite the sample bias inherent to CPG selection, the sample was adequate for the purposes of this study.

## Conclusions

The major contribution of this study is that it emphasizes the relevant role that laboratory medicine specialists and scientific societies play in the dissemination of scientific information by fostering collaboration between panels of experts that recommend CPGs which include relevant information to each particular field of knowledge. We recommend laboratory medicine specialists to reinforce their role as consultants, foster interdepartmental cooperation, and draw attention to how a clinical laboratory works, to avoid errors and improve patient care [36]. Laboratory professionals are responsible for ensuring that all phases of laboratory testing are performed appropriately, from the preanalytical and postanalytical phase where most errors concentrate, and which specialists and patients are less aware, through the analytical phase, where errors are less frequent (13–32%) [37].

Moreover, this study draws attention to points of improvement in the development of CPGs. The development of a CPG is an example of an interdisciplinary project where laboratory specialists can cooperate and share their expertise. The involvement of laboratory medicine specialists in interdisciplinary projects raises awareness about their relevant roles and functions. Further studies are needed to investigate the level of implementation of CPGs in daily laboratory practice, assess their impact on healthcare quality, and evaluate laboratory professionals’ involvement in the development and application of CPGs.
